# Genetically predicted effects of physical activity and sedentary behavior on myasthenia gravis: evidence from mendelian randomization study

**DOI:** 10.1186/s12883-023-03343-y

**Published:** 2023-08-11

**Authors:** Jiao Li, Fei Wang, Chen Zhang, Zhen Li, Juan Gao, Haijie Liu

**Affiliations:** 1 Department of Neurology, Xuanwu Hospital, Capital Medical University, No. 45 Changchun Street, Xicheng District, Beijing, 100053 China; 2 Department of Neurology, PLA Rocket Force Characteristic Medical Center, No. 16 Xinjiekouwai Street, Xicheng District, Beijing, 100088 China; 3 Department of Neurology, Central Hospital, Baoding No. 1, Baoding, 071000 China

**Keywords:** Genome-wide association studies, Mendelian randomization, Myasthenia gravis, Physical activity, Sedentary behavior

## Abstract

**Background:**

Myasthenia gravis (MG) is an autoimmune disorder affecting the neuromuscular junction. Despite the potential benefits of higher physical activity and lower sedentary behavior in MG patients, evidence from observational studies for the effect of physical activity on the risk of MG is limited and inconclusive.

**Methods:**

We employed linkage disequilibrium score (LDSC) regression, two-sample Mendelian randomization (MR), and its multivariable extension analyses (MVMR) to assess the relationship between leisure screen time (LST), moderate-to-vigorous intensity physical activity during leisure time (MVPA) and the risk of MG using genome-wide association studies (GWAS) summary datasets. MR analyses were performed using the inverse-variance-weighted (IVW), weighted-median, and MR-Egger regression. Sensitivity analyses were further performed using alternative instruments to test the robustness of our findings.

**Results:**

We found evidence of genetic overlap between LST (rg = 0.113, P = 0.023) and MG, as well as between MVPA (rg=-0.220, P = 0.0001) and MG, using LDSC method. The results of the MR suggested an association between genetic liability to LST and increased risk of MG (IVW OR = 1.609, 95% CI = 1.153 to 2.244; P = 0.005). This association was particularly notable for late-onset MG (IVW OR = 1.698, 95% CI = 1.145 to 2.518; P = 0.008), but not for early-onset MG. Consistent findings were obtained in the MVMR analysis using BMI as covariate (IVW OR = 1.593, 95% CI 1.167 to 2.173, P = 0.003). However, the MR analysis does not support a substantial causal effect of MVPA on the risk of MG.

**Conclusion:**

Our findings support a causal effect of sedentary behavior as measured by LST on MG, indicating that lack of exercise may play a role in the development of MG. Longitudinal and interventional studies of this association are warranted.

**Supplementary Information:**

The online version contains supplementary material available at 10.1186/s12883-023-03343-y.

## Background

Myasthenia gravis (MG) is a complex neurological disorder influenced by both genetic and environmental factors [1] and characterized by weakness of the skeletal muscles [2]. It is caused by autoantibodies that bind to functionally important molecules at the postsynaptic membrane at the neuromuscular junction [3]. Eighty per cent of patients with MG have detectable antibodies against the acetylcholine receptor (AChR), whereas a small minority instead have antibodies against muscle-specific kinase (MuSK) or lipoprotein-receptor-related protein 4 (LRP4) [3]. Nevertheless, the underlying factors contributing to the development of MG are not well understood [1]. The estimated heritability of MG is approximately 25.6%, which suggests there are genetic factors contributing to the disease that have yet to be identified [4]. In addition, the concordance of monozygotic MG twins is estimated to be about 35%, suggesting the important role of environmental variables in MG pathogenesis [5]. However, few lifestyle risk factors have been identified that are associated with the onset or progression of MG [6].

Physical activity (PA) is broadly defined as musculoskeletal movement that leads to energy expenditure [7]. There is growing interest in the potential of physical activity to preserve immunity and protect against multi-morbidity [8]. Conversely, excessive sedentary behavior has negative impacts on health regardless of other factors, suggesting that physical activity and sedentary behavior and may act as two independent risk factors predisposing individuals to poor health outcomes [9]. Despite several studies have evaluated the potential benefits of higher physical activity and lower sedentary behavior in MG patients [10–13], the relationship between PA and the risk of MG remains inconclusive due to the limited availability of longitudinal data.

Mendelian randomization (MR) overcomes these difficulties by using genetic variants associated with an exposure as instrumental variables (IVs) to inter the causality of the risk factor with respect to disease [14]. Leveraging the most recent GWAS datasets [15, 16], we employed linkage disequilibrium score (LDSC) regression, bidirectional MR, and its multivariable extension analyses (MVMR) to assess the relationship between leisure screen time (LST), moderate-to-vigorous intensity physical activity during leisure time (MVPA) and the risk of MG. Additionally, we explored the potential pleiotropic function of body mass index (BMI) [17], a notable confounder in research involving PA and sedentary behavior, in this interplay.

## Methods

### Study design

We performed this study under a two-sample, bidirectional, MR analysis framework. The MR approach we used was based on the following three assumptions: (1) genetic variants (single nucleotide polymorphisms (SNPs)) used as IVs are associated with exposures; (2) genetic variants are not associated with confounders; and (3) genetic variants influence the risk of outcomes only through interested exposures, not through other pathways. To begin, we performed a genetic correlation analysis using LDSC method to assess the shared genetic risks between LST and MVPA with MG. Next, we examined whether there was a bidirectional association between LST, MVPA, and the risk of MG. Finally, we conducted multivariable MR analysis to explore the potential mediation effect of body mass index (BMI) on the association between LST, MVPA, and the risk of MG.

### Linkage disequilibrium score (LDSC) regression

Linkage Disequilibrium Score (LDSC) Regression (https://github.com/bulik/ldsc) was employed to evaluate the genetic correlation across features to investigate the possibility of a shared genetic architecture [18]. Precomputed LD scores of European individuals in the 1000 Genomes project for high-quality HapMap3 SNPs were used (‘eur_w_ld_chr’). Using LDSC regression, we estimated genetic correlation (rg) between two traits by incorporating LD scores and GWAS summary statistics in a regression model.

### Mendelian randomization (MR) analyses

For selecting the most unbiased and representing instrumental genetic variables, we included SNPs associated with LST, MVPA and BMI at genome-wide significance (p < 5 × 10^− 8^). Then the index SNPs were obtained by clumping all significant SNPs within each linkage disequilibrium (LD) block from all GWAS using an R^2^ < 0.001 threshold (clumping window 10,000 kB). To prevent the effect estimates from aligning with different alleles, harmonization was performed to remove ambiguous SNPs showing non-concordant alleles. A more conservative strategy was performed to correct the strand for non-palindromic SNPs and drop all palindromic SNPs from the MR analysis. Overall, 91 instruments for LST and 12 instruments for MVPA were used in the two-sample MR analysis. The resulting lists of instrument SNPs for all phenotypes are provided in Supplemental Tables 2 and Supplemental Table 4.

The primary analyses were performed by the IVW method (hereafter referred to as standard MR analysis), which provides reliable estimates assuming all SNPs meet the IV assumptions and biases if average pleiotropic effect differs from zero. Then, the analyses were conducted utilizing the weighted median approach [19] and the MR-Egger regression method [20]. The weighted median regression calculates the effects that are robust if half or more of the SNPs are valid instruments, while the MR-Egger regression only relies on the instrument strength independent of direct effect assumption and not additionally on non-zero mean pleiotropy. Additionally, Cochran’s Q test and MR-Egger intercept test were utilized to assess the heterogeneity between causal estimates from different genetic variants, which can detect the presence of pleiotropy. The average pleiotropic effect of the genetic variants could be estimated by the intercept term (tested here using a p-value threshold of < 0.05). Besides, the leave-one-out analysis was conducted to assess the influence of potentially pleiotropic SNPs on the causal estimates. The multivariable MR analyses, in which BMI may potentially mediate the link across LST and MVPA with MG, were conducted similarly. Statistical analyses were carried out using the TwoSampleMR R package.

## Results

### Data sources

The data sources were selected from studies with publicly available GWAS summary data, and detailed information regarding the various GWAS datasets is displayed in Table 1. We extracted summary data from the most recent and largest meta-GWAS of physical activity which initially included four self-reported PA or sedentary behavior related traits: MVPA, leisure screen time (LST), sedentary commuting and sedentary behavior at work, and yield 88 loci associated with LST, 11 loci for MVPA, 4 loci for sedentary behavior at work, and no loci were identified for sedentary commuting [15]. To ensure adequate statistical power in instrumental variable and enrichment analyses, sedentary behavior at work and sedentary commuting were not included in the further post-GWAS analysis in the original dataset. Therefore, we also utilized LST (n = 526,725) and MVPA (n = 608,595) in our study. MG GWAS Summary statistics involving 1,873 patients versus 36,370 age/gender-matched controls collected from US and Italian [16] were used. To specify the age-dependent genetic heterogeneity of MG, we drew on summary statistics of early-onset (EOMG) (595 patients versus 2,718 controls, aged 40 years or younger) and late-onset (LOMG) (1,278 patients versus 33,652 controls) separately [16]. We also investigated the potential pleiotropic role of BMI in this interplay and GWAS summary of BMI which included 806,834 European ancestry individuals was retrieved from the GIANT consortium [17].

**Table 1:** Summary of the source GWAS datasets used in LDSC and MR.

Traits	First Author (Year)	Sample size (case/control)	Population	Phenotype ascertainment	GWAS Catalog accessions
LST	Zhe Wang (2022)	526,725	European	Time spent watching TV, playing videogames, and sitting at the computer, etc.	GCST90104339
MVPA	Zhe Wang (2022)	608,595	European	Swimming and Jogging, etc. Binary into “Active” vs. “inactive”	GCST90104341
BMI	Sara L Pulit (2019)	806,834	European	Meta-analysis of BMI in UK Biobank and GIANT data. Combined set of samples.	GCST009004
MG	Ruth Chia (2022)	38,243 (1,873/36,370)	US and Italy	Diagnosed based on standard clinical criteria and electrophysiological and/or pharmacological abnormalities, confirmed by the presence of anti-AChR antibodies.	GCST90093061
EOMG		3,313 (595/2,718)		EOMG ≤ 40 years old	GCST90093465
LOMG		34,930 (1,278/33,652)		LOMG > 40 years old	GCST90093466

BMI: body mass index; LST, Leisure screen time; MVPA, Moderate-to-vigorous intensity physical activity during leisure time; MG, myasthenia gravis; EOMG, early-onset myasthenia gravis; LOMG, late-onset myasthenia gravis

### LDSC regression demonstrates a correlation between PA and MG

Using LDSC regression, we found a significant positive correlation between LST and MG (rg = 0.1134, P = 0.0230), as well as a significant negative correlation between MVPA and MG (rg = -0.2202, P = 0.0001) with both MG subtypes (Table 2). Furthermore, BMI was also significantly positively correlated with MG (rg = 0.1010, P = 0.0096) and LOMG (rg = 0.1129, P = 0.0046) **(Supplemental Table 1)**. These novel findings support the genetic link hypothesis between PA and sedentary-related characteristics and MG.

**Table 2:** Pairwise genetic correlations between PA-related traits and MG using LD score regression

Traits	MG			EOMG			LOMG	
	**rg**	***P*** **value**		**rg**	***P*** **value**		**rg**	***P*** **value**
LST	0.1134	0.0230		0.0824	0.2335		0.0969	0.0626
MVPA	-0.2202	0.0001		-0.2202	0.0001		-0.1985	0.0004

### MR analysis show LST was associated with increased risk of MG

**Fig. 1:**
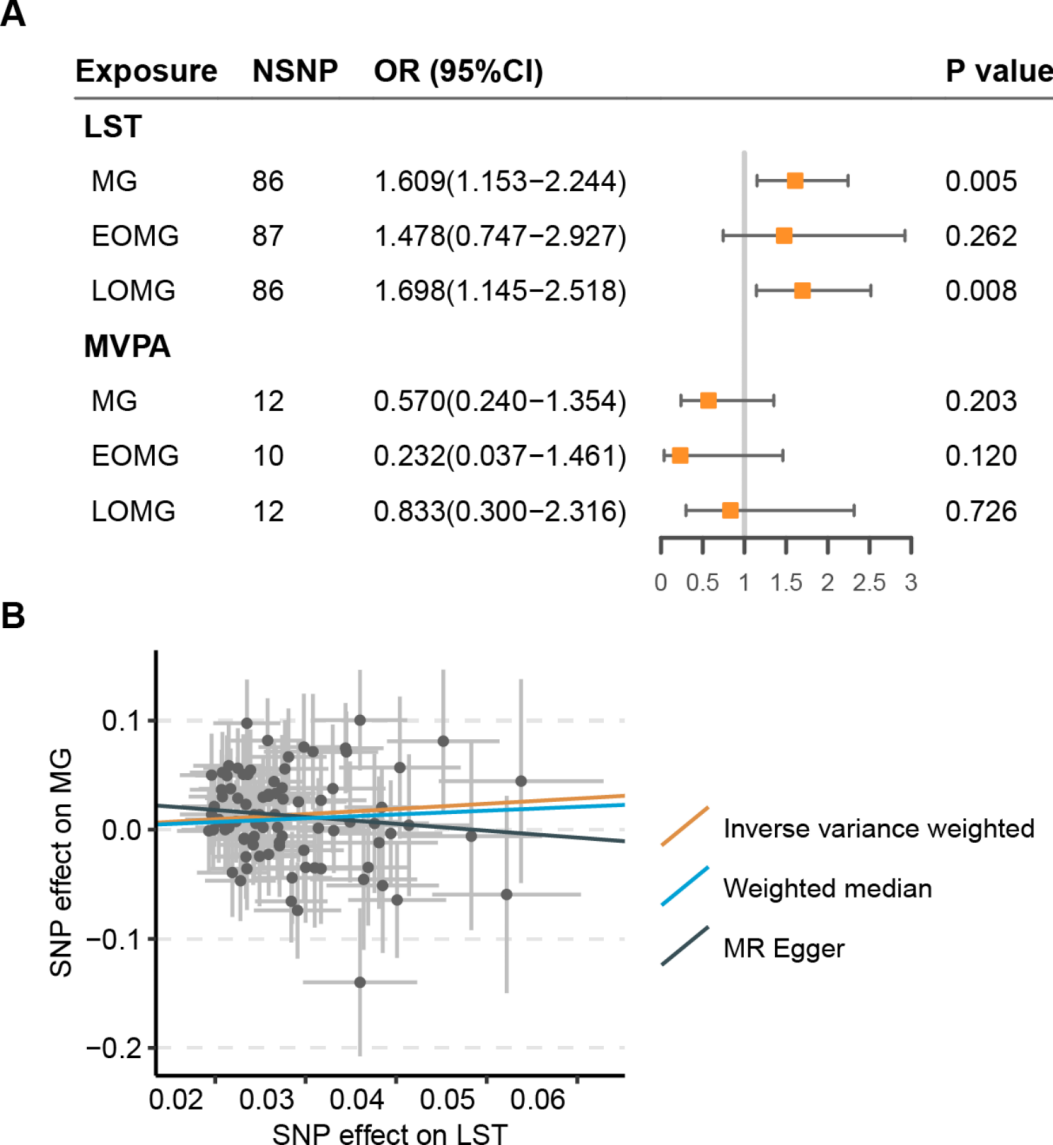
Univariant MR analysis for the effect of physical activity and sedentary-related traits on MG. **(A)** Forest plot of univariable MR-estimated effects sizes by the inverse-variance-weighted method. Data are expressed as OR values with 95% CI. LST, Leisure screen time; MVPA, Moderate-to-vigorous intensity physical activity during leisure time; MG, myasthenia gravis; EOMG, early-onset myasthenia gravis; LOMG, late-onset myasthenia gravis. *P* values in bold indicates *P* < 0.05/2. **(B)** Scatterplot of SNP potential effects on LST vs. MG, with the slope of each line corresponding to estimated MR effect per method

The results of the MR suggested an association between LST and increased risk of MG (IVW OR = 1.609, 95% CI = 1.153 to 2.244; P = 0.005), and late-onset MG (IVW OR = 1.698, 95% CI = 1.145 to 2.518; P = 0.008). However, both weighted median and MR Egger analysis yielded a pattern of no effects. In addition, the MR estimate was not statistically significant when compared to LST and EOMG (IVW OR = 1.478, 95% CI = 0.747 to 2.927; P = 0.262). The causal relationships between MVPA and MG were not statistically significant (IVW OR = 0.570, 95% CI = 0.240 to 1.354; P = 0.203) (Fig. 1). The results of sensitivity analyses using Cochran’s Q test and MR-Egger intercept test suggested that there was no heterogeneity or horizontal pleiotropy among the SNP effect estimates **(Supplemental Tables 3 and Supplemental Fig. 1)**. In addition, a leave-one-out analysis was conducted to examine the influence of pleiotropic genetic variants, however we did not identify a single genetic variant that affected the association between LST with MG and LOMG (**Supplemental Fig. 2**).

Reverse MR also did not demonstrate any causal effect of MG on LST (IVW OR = 0.991, 95% CI = 0.973 to 1.008; P = 0.301) and MVPA (IVW OR = 0.997, 95% CI = 0.986 to 1.009; P = 0.643). However, Cochran’s Q test of MG on LST (Q = 15.739; P = 0.015) suggested the possibility of heterogeneity SNP(s) in our dataset **(Supplemental Table 3)**.

Since there is some evidence that levels of physical activity and BMI have a genetic association [15], we employed a multivariable MR approach for LST and MVPA with BMI as a confounding variable **(Supplemental Table 4)**. Consistent with the results of univariate MR analyses that did not include BMI as a covariate, we found evidence confirming a causal association between LST with BMI against MG (OR = 1.593, 95% CI 1.167 to 2.173, *P* = 0.003), and LOMG (OR = 1.771, 95% CI 1.216 to 2.580, *P* = 0.003), but not EOMG. In contrast, the association between MVPA with BMI versus MG and MG subtypes was not significant after Bonferroni correction (Table 3).

**Table 3:** Multivariable MR results using significant loci only

Exposure	Outcome	NSNP	Beta	SE	*P* value	OR (95%CI)
LST BMI	**MG**	97	0.465	0.159	0.003	1.593(1.167–2.173)
	**EOMG**	97	0.096	0.323	0.767	1.101(0.584–2.074)
	**LOMG**	97	0.572	0.192	0.003	1.771(1.216–2.580)
MVPA BMI	**MG**	11	-0.652	0.285	0.022	0.521(0.298–0.911)
	**EOMG**	11	-0.412	0.554	0.457	0.662(0.223–1.963)
	**LOMG**	11	-0.512	0.347	0.140	0.599(0.304–1.182)

## Discussion

This is the first study to examine genetic correlations and causal associations across GWAS datasets between physical activity, sedentary behavior-related characteristics, and the risk of MG. Our analysis using the LDSC method revealed a significant relationship between LST and MVPA in relation to MG. Then, we applied MR analysis based on genetic instruments selected from the most recent large-scale GWASs, which suggested a causal relationship between LST and MG, independent of whether BMI was a confounder. Consistent with the prior research [1, 2], the generic difference between EOMG and LOMG was observed that only the LOMG had a significant association with LST in MR analysis.

The benefits of physical exercise for healthy individuals are well-established, particularly in terms of mitigating the risks of chronic lifestyle-related diseases [21]. PA can generate an anti-inflammatory response by significantly boosting T-regulatory cells, reducing immunoglobulin secretion and promoting the release of IL-6 from muscles [7] A recent study also utilized MR analysis and identified T-cell traits as causally protective factors for MG [22]. Therefore, PA is considered a protective factor for certain autoimmune disorders, including rheumatoid arthritis [23] and multiple sclerosis [24], which are also genetically associated with MG [15]. In the case of MG, previous studies indicate that lower levels of PA have a detrimental effect on muscle mass and strength [25, 26] and that PA restriction is a predictor of fatigue severity [12, 27]. Thus, the genetic liability of PA and MG is uncertain to distinguish if PA causally influences risk for MG, or whether there is reverse causality due to fatigue and reduction of PA in the prodromal phase. In our results, despite the association between MVPA and MG observed in LDSC, the univariable and multivariable MR analyses do not suggest a substantial causal effect of MVPA on the risk of MG. However, as the number of genetic variants for the MVPA trait is modest, resulting in low statistical effectiveness, our MR analyses may not be currently powered to detect weak effects.

Our results suggest a moderate causal relationship between genetic susceptibility to LST and MG. The LST was defined as leisure time in front of screens, mostly about TV, computer, and video games, which is generally considered a physical inactivity lifestyle. Physical inactivity can activate inflammatory pathways and lead to elevated serum/plasma concentrations of inflammatory markers, such as IL-6, IL-1β and TNF-α [28]. Recent research identified LST-associated genes that are enriched in skeletal muscle and altered by resistance training, some of which pointed to myopathy and T cell-related pathways [15]. This provides genetic insights into how complex traits such as sedentary behaviors are associated with MG. However, the complex and multifactorial association between MG and physical activity cannot be fully explored solely through MR analysis. Therefore, additional epidemiological evidence is necessary to establish causality. We approach the determination of LST as a potential causal risk factor for MG with caution.

## Limitations

Several limitations merit consideration in our study. First, although we included data from the largest GWAS dataset on PA and sedentary behaviors, we are unable to carry out a more extensive analysis due to the lack of genetic variables for heterogenous PA or sedentary behavior-related factors, especially the MVPA traits which are just dichotomized in GWAS. Second, despite the selection of SNPs with strong associations, common SNPs cannot be considered exact proxies for exposure since they do not adequately explain the overall variance in complex traits. Thirdly, all the GWAS data were extracted primarily from European ancestry; hence, additional research from other ethnicities is required to bolster the conclusions.

## Conclusions

Our findings support a causal effect of sedentary behavior as measured by LST on MG, indicating that lack of exercise may play a role in the development of MG; however, longitudinal and intervention studies are needed to determine the relationship.

### Electronic supplementary material

Below is the link to the electronic supplementary material.

Supplementary Material 1

Supplementary Material 2

Supplementary Material 3

## Data Availability

The original contributions generated for this study are included in the article, and further inquiries can be directed to the corresponding author.
