# Supplementary figures and images for: Genetically predicted effects of physical activity and sedentary behavior on myasthenia gravis: evidence from mendelian randomization study
Source: BMC Neurol. 2023 Aug 11;23:299. doi: 10.1186/s12883-023-03343-y (PMC10416521; doi:10.1186/s12883-023-03343-y)

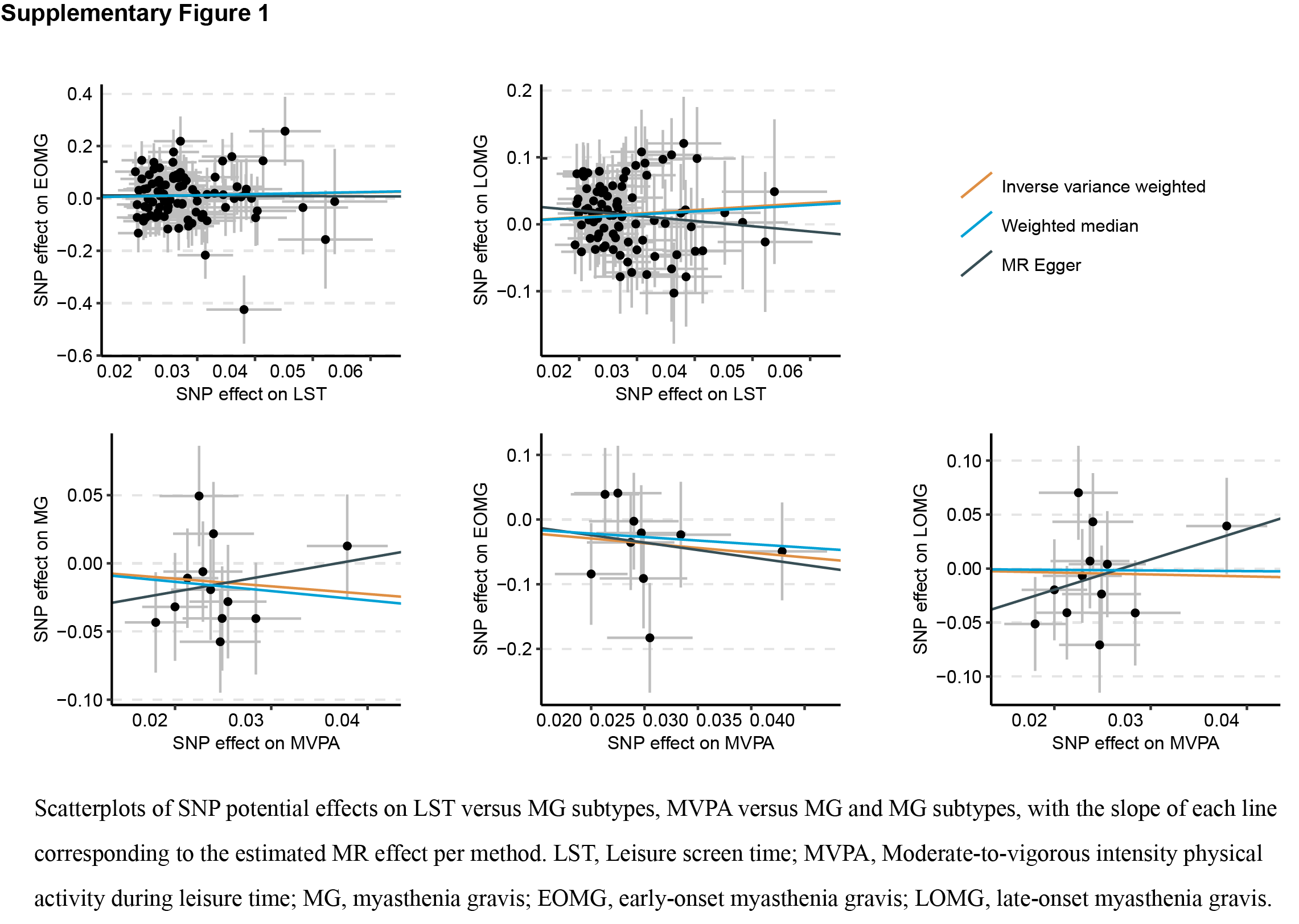

Supplement: Supplementary file 2 — Supplementary Material 2 [file 12883_2023_3343_MOESM2_ESM.tif]

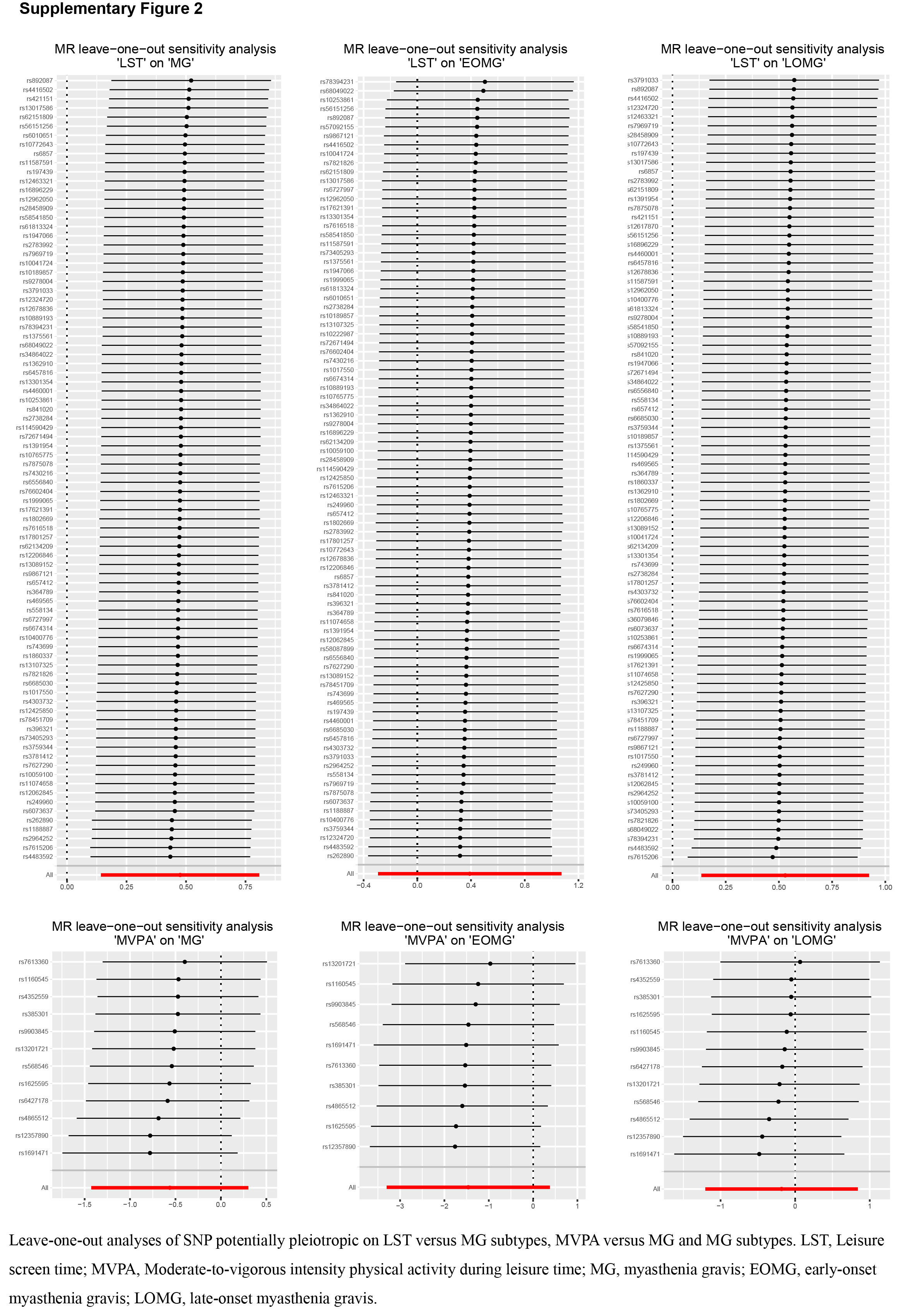

Supplement: Supplementary file 3 — Supplementary Material 3 [file 12883_2023_3343_MOESM3_ESM.tif]
